# Widely Targeted Metabolomics Reveals Metabolic Divergence in *Abutilon theophrasti* Populations Under Glufosinate Ammonium Treatment

**DOI:** 10.3390/plants14131994

**Published:** 2025-06-30

**Authors:** Xiaotong Guo, Yu Wang, Yulian Guo, Chan Luo, Keqiang Cong

**Affiliations:** Institute of Plant Protection, Heilongjiang Academy of Agricultural Sciences, Harbin 150086, China; xtg96318@163.com (X.G.); wyhrb71@163.com (Y.W.); luochan1984528@126.com (C.L.); 18846916169@163.com (K.C.)

**Keywords:** *Abutilon theophrasti*, glufosinate ammonium, widely targeted metabolomics, metabolic pathways

## Abstract

*Abutilon theophrasti* Medikus, a pervasive weed infesting transgenic corn fields, exhibits increasing tolerance to glufosinate ammonium—a widely used herbicide in genetically modified cropping systems. This study employed a widely targeted metabolomics approach to investigate differential metabolic responses to glufosinate ammonium across two *Abutilon theophrasti* populations under identical treatments. A total of 2546 metabolites were detected, predominantly classified into alkaloids, amino acids and derivatives, and flavonoids, among other categories. Three pivotal metabolic pathways (Arginine and proline metabolism, Biosynthesis of amino acids, D-amino acid metabolism) were identified as critical regulators of herbicide response. These findings advance our understanding of weed metabolic adaptation to glufosinate ammonium and lay a foundation for elucidating potential herbicide resistance mechanisms in weeds.

## 1. Introduction

*Abutilon theophrasti* Medikus, an annual weed of the genus *Abutilon*, is recognized as one of the most pernicious weeds in various cropping systems across China. Its aggressive growth patterns, high reproductive coefficient, and remarkable adaptability enable intense competition with crops for light, water, and nutrients, resulting in substantial yield reductions under co-occurring conditions [1,2].

Research has demonstrated that high-density infestations of *Abutilon theophrasti* (≥12 plants/m^2^) can induce severe yield reductions in maize, potentially leading to near-complete yield loss [1,2]. Furthermore, *Abutilon theophrasti* exhibits pronounced biological characteristics, including exceptional fecundity and prolonged seed viability in soil seed banks, rendering complete eradication challenging and perpetuating persistent agricultural threats [3]. As a widely distributed weed species across various regions, *Abutilon theophrasti* typically germinates between April and July with seed reproduction as its primary propagation mechanism [4]. Current management strategies predominantly rely on herbicidal control. However, recent alterations in agricultural practices and over-reliance on monoactive herbicides have accelerated weed community succession. Consequently, *Abutilon theophrasti* infestation dynamics have exhibited escalating trends, progressively manifesting as a pernicious weed in maize ecosystems. While herbicide application has significantly mitigated weed-induced crop damage, the agricultural sector now confronts emerging challenges stemming from herbicide resistance evolution. Intensive and repeated application of herbicides with similar modes of action has been selected for herbicide-resistant weed biotypes, consequently depreciating chemical control efficacy and exacerbating weed management complexities. Notably, populations of atrazine-resistant *Abutilon theophrasti* biotypes have been documented in corn and soybean production systems as of 2025 [5].

Current weed management still predominantly relies on chemical control methods. However, persistent herbicide applications have induced multiple ecological challenges in agricultural ecosystems, including resistance development, spray drift, leaching contamination, and biodiversity reduction. Among these, herbicide resistance remains the most critical agricultural issue, leading to diminished weed control efficacy, increased production costs, and progressive replacement of susceptible biotypes by resistant variants [6].

Glufosinate ammonium (GA), a broad-spectrum, non-selective, low-toxicity organophosphorus herbicide, exhibits effective control against diverse weed species [7,8]. Structurally analogous to glutamic acid, GA exerts its herbicidal action through irreversible inhibition of glutamine synthetase (GS) (EC 6.3.1.2) activity—an enzyme essential for ammonia detoxification in weeds—thereby disrupting photosynthetic processes [9]. GS ranks as the second most abundant protein in plant leaves and serves as a critical enzyme for nitrogen assimilation, metabolism, and photorespiration. GA-mediated GS inhibition induces reactive oxygen species (ROS) accumulation [8,10].

Due to limited translocation within plants, GA primarily delivers contact toxicity through foliar absorption, with phytotoxic effects localized to treated surfaces [11]. Characteristic symptoms manifest as leaf chlorosis followed by rapid plant mortality, typically occurring within 2–5 days post-application [12]. The rapid phytotoxicity of GA has been attributed to ammonia accumulation in foliar tissues [8]. Emerging evidence suggests ROS may serve as primary mediators of GA’s acute toxicity [10]. Metabolic rate variations in plant GA processing have been reported to correlate with differential sensitivity or resistance phenotypes to this compound [13,14].

Glufosinate ammonium possesses distinctive physicochemical properties compared to most herbicides currently used worldwide, representing the most significant glutamine synthetase (GS)-targeting herbicide. As is well established, continuous application of herbicides with identical modes of action intensifies selection pressure for resistance development in weed populations [15]. Physiological adaptations conferring herbicide resistance typically involve reduced herbicide absorption, vacuolar sequestration, altered membrane transport activity, modified translocation patterns, and enhanced metabolic detoxification [15]. Among these mechanisms, reduced absorption constitutes the primary resistance pathway developing in initially susceptible weed biotypes [16]. Vacuolar sequestration refers to the plant’s ability to compartmentalize applied herbicides within cellular vacuoles or trichomes, thereby inhibiting their translocation [17]. Glyphosate sequestration has been extensively documented in various weed species, including *Eleusine indica* (L.) Gaertn., *Lolium rigidum* Gaudin., and *Conyza canadensis* (L.) Cronquist [18,19]. In *Conyza canadensis*, rapid glyphosate absorption occurs post-application, with resistant biotypes exhibiting 85% vacuolar compartmentalization within 24 h, contrasting with only 15% in susceptible populations [19].

Currently, glyphosate and glufosinate ammonium remain the predominant broad-spectrum herbicides applied in transgenic crop fields across numerous countries [8,20]. Global utilization of glufosinate ammonium has demonstrated significant growth, particularly for weed management in glyphosate-resistant transgenic cropping systems [21]. Given the escalating prevalence of herbicide-resistant weeds, glufosinate ammonium plays a pivotal role in contemporary weed management strategies. The expanding development of multiple herbicide-resistant transgenic crops suggests sustained growth in glufosinate ammonium applications. To date, five weed species from Malaysia, the United States, and New Zealand have evolved resistance to glufosinate ammonium [5]. Nevertheless, the underlying resistance mechanisms remain insufficiently characterized, necessitating further empirical investigation.

During growth and development, plants encounter various biotic and abiotic stresses, orchestrating metabolic network reconfigurations to maintain essential metabolic processes and establish novel homeostatic states for environmental adaptation [22]. The remodeling of metabolomes under stress conditions fundamentally reflects plants’ adaptive responses and defense mechanisms, with metabolomics serving as a robust analytical platform for investigating stress-induced metabolic reprogramming. Metabolomics enables comprehensive qualitative and quantitative analysis of all low-molecular-weight metabolites within defined biological systems through standardized protocols [23]. This discipline aims to integrate complex multi-dimensional datasets by combining advanced analytical platforms with sophisticated biostatistical strategies, thereby elucidating dynamic metabolic flux variations and pathway regulatory mechanisms [24].

Contemporary applications of metabolomics span diverse plant research domains, including phenotypic characterization, breeding assistance, bioactive compound profiling, and mechanistic investigations into stress resistance [25]. This high-throughput approach facilitates rapid quantification of accumulated phytochemicals while revealing intrinsic relationships between metabolic signatures and genetic/environmental perturbations.

Differential metabolite profiling under stress conditions provides critical theoretical insights into three key aspects: (1) stress-specific accumulation patterns of specialized metabolites, (2) pathway-level metabolic redirection mechanisms, and (3) systemic regulatory networks underlying stress tolerance. Current implementations of metabolomics encompass comparative analyses across species, tissues, and stress conditions [26,27]. Widely targeted metabolomics merges the broad-spectrum coverage of non-targeted approaches with the precision of targeted analyses, utilizing multiple reaction monitoring (MRM)-based data acquisition and established metabolite databases for enhanced identification accuracy and quantification reliability [28]. This hybrid methodology combines a high throughput with precise compound annotation, overcoming limitations inherent to conventional metabolomic workflows.

## 2. Results

### 2.1. Results of the Whole-Plant Bioassay

**Figure 1:**
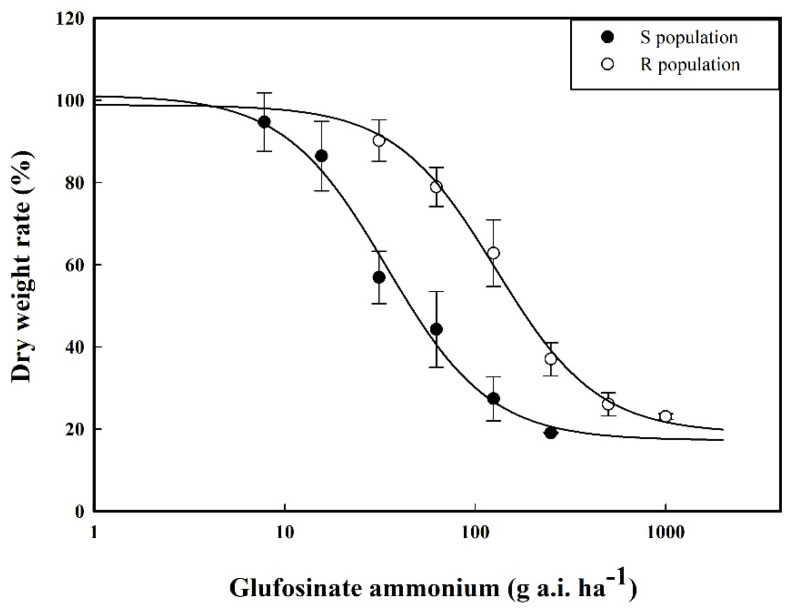
The dose-response curves of *Abutilon theophrasti* populations to glufosinate ammonium.

The whole-plant bioassay results demonstrated that the GR_50_ value of the susceptible (S) *A. theophrasti* population was 34.44 g a.i. ha^−1^, while that of the resistant (R) population reached 128.53 g a.i. ha^−1^, representing a 3.73-fold resistance ratio compared to the S population (Figure 1). These findings revealed statistically significant differences in GR_50_ values between the two *A. theophrasti* populations.

### 2.2. Overview of the Nonvolatile Metabolites of Abutilon theophrasti

**Figure 2:**
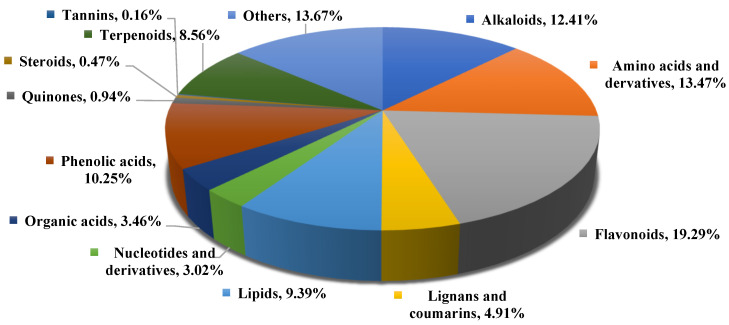
The pie chart of the number of different types of metabolites.

Utilizing the UPLC-MS/MS detection platform combined with a self-built database (Metware database), a total of 2546 metabolites were detected across 4 treatment groups of *Abutilon theophrasti* (Figure 2), including 316 alkaloids, 343 amino acids and derivatives, 491 flavonoids, 125 lignans and coumarins, 239 lipids, 77 nucleotides and derivatives, 88 organic acids, 261 phenolic acids, 24 quinones, 12 steroids, 4 tannins, 218 terpenoids, and 348 others (Table S1).

### 2.3. Metabolite Qualitative–Quantitative Analysis and Sample Quality Control

Mass spectrometry data were processed with Analyst 1.6.3 software. Figure S1A,B presented the total ion current (TIC) chromatogram of quality control (QC) pooled samples and the multiple reaction monitoring (MRM)-based metabolite detection multi-peak chromatogram (extracted ion chromatogram). Notably, the overlaid display analysis of TIC chromatograms from different QC samples confirmed excellent reproducibility in metabolite extraction and detection throughout this experiment, thereby providing crucial validation for data repeatability and reliability.

The coefficient of variation (CV), calculated as the ratio of the standard deviation to the mean value of raw datasets, is utilized as a principal metric for quantifying data dispersion. A higher proportion of metabolites with lower CV values in QC samples reflects greater experimental stability. When over 85% metabolites exhibited CV < 0.5, the dataset was considered stable, whereas over 75% metabolites with CV < 0.3 indicated exceptional experimental stability. Our results demonstrated robust data consistency, fulfilling these stringent quality criteria (Figure S2).

### 2.4. Principal Component Analysis (PCA) and Grouped Principal Component Analysis

**Figure 3:**
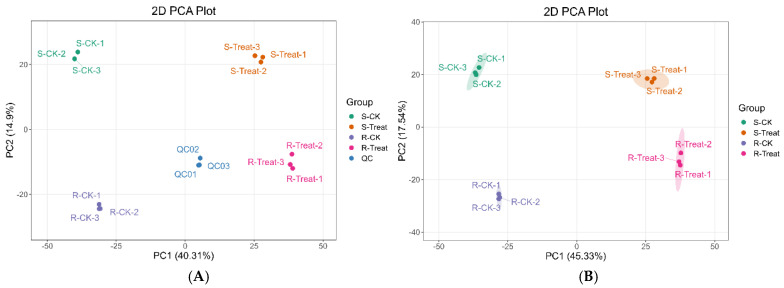
PCA score plot of mass spectrometry data for experimental groups and quality control (QC) samples ((**A**): with QC, (**B**): no-QC). Each datapoint in the figure corresponds to an individual biological sample, with color uniformity indicating group membership. PC1 represents the first principal component, PC2 represents the second principal component and the percentage indicates the interpretation rate of this principal component for the dataset.

Principal Component Analysis (PCA), an unsupervised pattern recognition methodology employing multivariate statistics, is fundamentally conducted through an orthogonal transformation process. Within this procedure, potentially correlated variables are systematically converted into linearly uncorrelated principal components. PCA was performed on samples to preliminarily characterize the overall metabolite disparities between the experimental groups and intra-group variabilities. The PCA results revealed distinct separation trends in metabolomic profiles among groups, indicating the presence of metabolic heterogeneity within the sample groups [29]. The corresponding PCA score plot is presented below (Figure 3). Prior to differential analysis, PCA was initially conducted on samples from the compared groups to assess inter-group differences and intra-group variations.

### 2.5. Cluster Analysis

**Figure 4:**
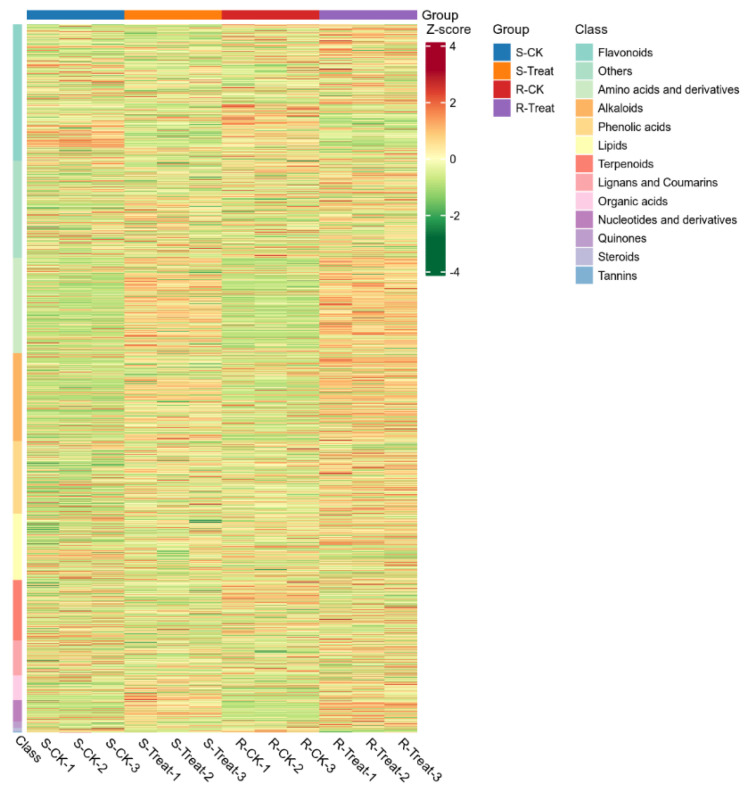
Heatmap of the changes in R-Treat, S-Treat, R-CK and S-CK samples under different treatment.

Cluster analysis constitutes a multivariate statistical classification methodology that partitions objects into categories characterized by maximal intra-class homogeneity and inter-class heterogeneity. Metabolite concentration data were processed using unit variance scaling (UV). Hierarchical cluster analysis (HCA) was subsequently performed via the ComplexHeatmap package in R software (2.9.4) to visualize metabolite accumulation patterns across samples through heatmap construction. The cluster analysis results for all samples were graphically represented using R-generated heatmaps (Figure 4 and Figure S3). The results revealed that 14 classes of metabolites were identified to undergo significant alterations across the samples. Specifically, metabolites including amino acids and derivatives, alkaloids, phenolic acids, nucleotides and derivatives, and quinones showed significantly higher abundances in the R-Treat group compared with the other three treatment groups. Relative to R-CK and S-CK groups, elevated levels of amino acids and derivatives, alkaloids, phenolic acids, organic acids, nucleotides and derivatives, and quinones were observed in the S-Treat and R-Treat groups. The R-CK and S-CK groups exhibited significantly increased accumulations of terpenoids, tannins, and steroids.

### 2.6. Orthogonal Partial Least Squares-Discriminant Analysis (OPLS-DA) and Model Validation

Partial Least Squares-Discriminant Analysis (PLS-DA), a supervised pattern recognition multivariate statistical method, extracts components from independent variable matrix X and dependent variable matrix Y, and then computes correlations between these components. Compared to PCA, PLS-DA maximizes inter-group separation, facilitating the identification of differential metabolites. Orthogonal Partial Least Squares-Discriminant Analysis (OPLS-DA) integrates Orthogonal Signal Correction (OSC) with PLS-DA, decomposing X matrix information into Y-correlated and Y-orthogonal components to enhance differential variable screening by removing irrelevant variations. During OPLS-DA modeling, the X matrix was partitioned into predictive components (Y-correlated) and orthogonal components (Y-uncorrelated). Score plots generated from metabolomic data analysis visually demonstrated inter-group metabolic disparities [30]. The calculation results of the OPLS-DA models for different treatment groups in this study are shown in Tables S2–S5 (Figure S4A,B). Model performance was assessed using three predictive parameters: R^2^X, R^2^Y, and Q^2^. R^2^X and R^2^Y, respectively, quantify the model explanatory power for the X and Y matrices, while Q^2^ reflects the predictive capacity. Values approaching 1 denote superior model reliability, with *Q*^2^ > 0.5 indicating valid models and *Q*^2^ > 0.9 representing exceptional performance. In this study, *Q*^2^ was 0.936, representing exceptional performance. A threshold of *p* < 0.05 was deemed optimal for model acceptance. In this study, the threshold was *p* < 0.005.

### 2.7. VIP Plot, and Volcano Plot of Differential Metabolites

**Figure 5:**
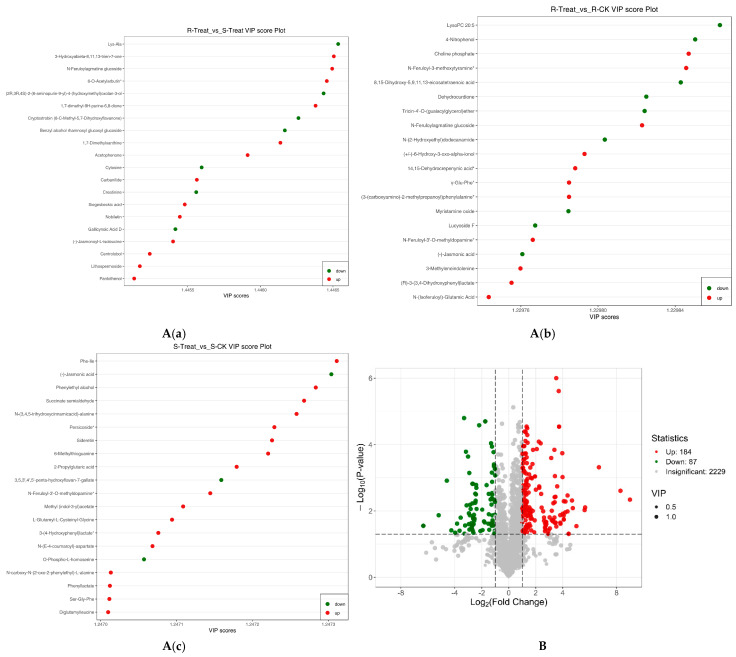
Top 20 compounds with highest VIP scores of R-Treat vs. S-Treat (**A**(**a**)). Top 20 compounds with highest VIP scores of R-Treat vs. R-CK (**A**(**b**)). Top 20 compounds with highest VIP scores of S-Treat vs. S-CK (**A**(**c**)). Volcano plot of the differential metabolites of R-Treat vs. S-Treat (**B**).

Metabolites meeting the screening criteria in the OPLS-DA models (Figure S5) were further prioritized by selecting the top 20 metabolites with highest VIP values for visualization (Tables S6–S8) [Figure 5A(a–c)].

A volcano plot was employed to simultaneously illustrate the relative abundance differences (log2 fold change) and statistical significance (−log10 transformed *p*-values) of the metabolites between pairwise groups (Figure 5B).

### 2.8. Metabolite Annotation and Metabolic Pathway Enrichment Analysis Result

**Figure 6:**
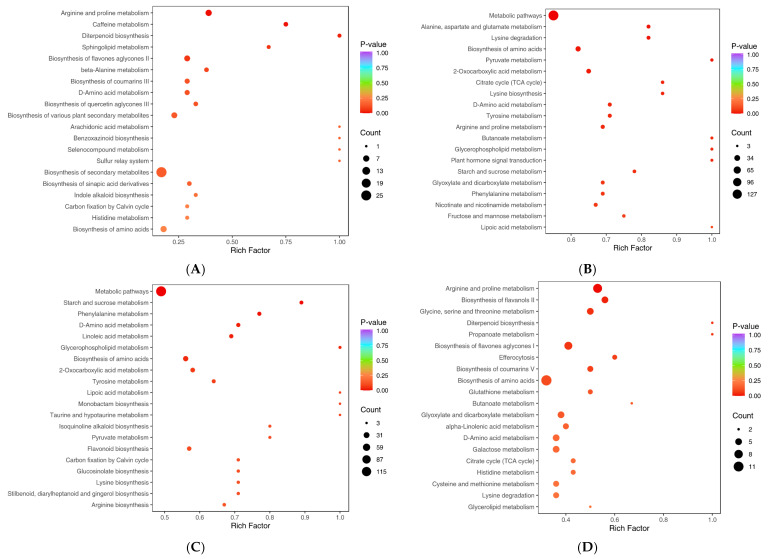
Enrichment map of differential metabolite pathways. The size of the points in the plot represents the number of significantly differential metabolites enriched in the corresponding pathway. The top 20 pathways ranked by P-value are displayed in ascending order ((**A**): R-Treat&S-Treat; (**B**): R-Treat& R-CK; (**C**): S-Treat&S-CK; (**D**): R-CK&S-CK).

Within biological systems, metabolites interact to form distinct pathways. Differential metabolites were functionally annotated via the KEGG database [31] and MetMap database, with the top 20 significantly altered metabolites categorized (Tables S9–S12) (Figures S6–S9) [Figure 5A(a–c)]. The bubble plot (Figure 6) for enrichment analysis was generated using hypergeometric distribution tests in R 4.1.2 to calculate the *p*-value for each pathway. Based on the differential metabolite results, pathway enrichment analysis was performed, in which the Rich Factor is the ratio of the number of differential metabolites in the corresponding pathway to the total number of metabolites annotated to that pathway. The higher value indicates a greater degree of enrichment. The *p*-value is the *p*-value from the hypergeometric test and the calculation formula for the hypergeometric distribution is shown below:

(1) P=1−∑i=0m−1MiN−Mn−iNn

Among them, *N* represents the number of metabolites possessing KEGG or MetMap annotations within all metabolites. *n* represents the number of differential metabolites in *N*. *M* represents the number of metabolites in a specific KEGG or MetMap pathway within *N*. *m* represents the number of differential metabolites in that specific KEGG or MetMap pathway within *M*. A *p*-value closer to zero indicates more significant enrichment. The size of the points in the plot represents the number of significantly differential metabolites enriched in the corresponding pathway. The top 20 pathways ranked by *p*-value are displayed in ascending order. After calculations, the bubble plot is generated using ggplot2 version 3.3.0. The major classes of differential metabolites included lipids, alkaloids, terpenoids, flavonoids, organic acids, phenolic acids, amino acids/derivatives, and nucleotides/derivatives. Notably, (-)-Jasmonic acid and *N*-Feruloyl-3′-O-methyldopamine were identified as shared metabolites between the R-Treat&R-CK and S-Treat&S-CK groups, suggesting their potential roles as key intermediates in glufosinate ammonium metabolism. *N*-Feruloylagmatine glucoside, common to the R-Treat&R-CK and R-Treat&S-Treat comparisons, may underlie differential glufosinate ammonium sensitivity between R and S populations. Pathway enrichment analysis was performed using the Rich Factor metric [(number of differential metabolites per pathway)/(total annotated metabolites in pathway)], where higher values indicate stronger pathway enrichment. The top 20 pathways ranked by ascending *p*-values are displayed (Figure 6A–D) (Figures S10–S13). The common metabolic pathways among the R-Treat&S-Treat, R-Treat&R-CK, S-Treat&S-CK, and R-CK&S-CK groups were identified as D-amino acid metabolism and Biosynthesis of amino acids. Shared metabolic pathways between R-Treat&S-Treat, R-Treat&R-CK, and R-CK&S-CK included Arginine and proline metabolism. The common metabolic pathways of R-Treat&S-Treat and R-CK&S-CK were Arginine and proline metabolism, Biosynthesis of amino acids, D-amino acid metabolism, and Histidine metabolism. The core metabolic pathways of R-Treat&S-Treat and R-Treat&R-CK were Arginine and proline metabolism, Biosynthesis of amino acids, and D-amino acid metabolism. Collectively, these findings indicate that herbicide metabolism in R and S populations predominantly involves Arginine and proline metabolism, Biosynthesis of amino acids, D-amino acid metabolism, and Histidine metabolism. However, metabolic divergence between R and S under glufosinate ammonium exposure appears to localize specifically to the Arginine and proline metabolism, Biosynthesis of amino acids, and D-amino acid metabolism pathways.

## 3. Discussion

The observed enrichment in D-amino acid metabolism may indicate their involvement in herbicide detoxification processes. Previous studies have established that specific D-amino acids can serve as precursors for secondary metabolites acting as chemical defenses in plants [32]. While the role of D-amino acids in herbicidal stress remains poorly characterized, their accumulation parallels findings in microbial systems where D-amino acids mediate detoxification through structural competition [33]. For instance, in bacteria, D-alanine enhances biofilm formation as a physical barrier against xenobiotics [34,35]. In plants, D-amino acids are increasingly recognized as signaling molecules under stress. Recent work demonstrated that D-serine modulates root cell wall lignification in *Arabidopsis* under aluminum toxicity through redox-modulated glutathione conjugation [36]. This may indicate that D-amino acids have a potential conservative role in structural defenses against glufosinate ammonium penetration. This mechanism shares similarities with phase II detoxification pathways described in herbicide metabolism [37]. This suggests a possible detoxification mechanism where specific D-amino acids facilitate herbicide conjugation, although their exact role in glufosinate ammonium metabolism requires further validation.

Glufosinate ammonium inhibits glutamine synthetase (GS), disrupting nitrogen assimilation and inducing intracellular ammonium hyperaccumulation, which triggers oxidative photodamage (ROS increase) [38,39]. The suppression of glutamine synthetase (GS) by glufosinate ammonium typically leads to ammonia accumulation. Upregulated biosynthesis may counteract nitrogen assimilation imbalances. This paradoxical pattern suggests compensatory upregulation of alternative nitrogen assimilation routes, such as the glutamate dehydrogenase (GDH) pathway—a well-documented bypass mechanism in glufosinate-resistant *Eleusine indica* [40]. Research found that reactive oxygen species trigger the rapid evolution of glufosinate resistance in *Lolium perenne,* and ROS regulatory networks may become new targets for inhibiting the evolution of resistance [38].

Proline acts as an osmoprotectant and ROS scavenger, and proline can directly remove hydroxyl radical (·OH) and singlet oxygen (^1^O_2_), improve the enzyme activities of CAT and POD, and enhance the ability of scavenging ROS [41,42]. Therefore, in the face of ammonium toxicity and oxidative stress induced by glufosinate ammonium stress, cell protection may be achieved through upregulation of the proline synthesis pathway. Arginine metabolism links to polyamine synthesis, which mitigates oxidative stress [43]. It has been observed that polyamines can stabilize membrane integrity under salt stress in rice [44]. Therefore, when GS activity is inhibited by glufosinate ammonium, plants may compensate for the imbalance of nitrogen metabolism and maintain redox homeostasis by activating polyamine synthesis. These may be compensatory responses to glufosinate-ammonium-induced oxidative damage. Oxidative damage caused by phosphine oxalammonium may trigger the accumulation of osmoregulatory substances (such as proline) and metabolic reprogramming of Arginine, alleviating ROS toxicity through polyamine synthesis [41,43]. From another point of view, the coordinated induction of the Arginine–proline–polyamine pathways likely delays cellular collapse, allowing for time for phase I/II detoxification systems (cytochrome P450s, GSTs) to process glufosinate—a temporal synergy critical for survival in low-dose herbicide exposure scenarios [45].

While this study pioneers the characterization of metabolic responses to glufosinate ammonium in *Abutilon theophrasti*, some noteworthy limitations should be acknowledged: (1) the observed metabolic changes require validation at transcriptional level; (2) the functional significance of specific metabolites remains to be elucidated through isotope tracing experiments; and (3) the quantification of the metabolites is only approximate because authentic standards were not used and detector sensitivity to metabolites often varies greatly.

## 4. Materials and Methods

### 4.1. Metabolite Annotation and Metabolic Pathway Enrichment Analysis

#### Plant Materials and Herbicides

Two *Abutilon theophrasti* populations were selected for this study: one (SR) collected from a field in Qiqihar with a three-year history of glufosinate ammonium application, and the other (S) from an uncultivated ditch edge with no herbicide exposure history.

A 200 g/L glufosinate ammonium aqueous solution (AS; registration code PD20110507) was purchased from Lier Crop Science Co., Ltd. (Mianyang, China).

### 4.2. The Whole-Plant Bioassay

#### 4.2.1. Plant Cultivation and Treatment

A 3:1 mixture of nutrient soil and vermiculite was homogenized and loaded into plastic pots (9 cm × 9 cm × 10 cm). *Abutilon theophrasti* seeds were surface-sterilized with 75% ethanol, rinsed five times with distilled water, soaked in distilled water for 12 h, and then sown. The plants were irrigated via sub-irrigation and thinned to eight uniform seedlings per pot at the two-leaf stage to minimize mutual shading.

Glufosinate ammonium was applied at varying doses (Table 1) during the 3–4-true-leaf stage, with three replicates per treatment. Herbicide applications were performed using an ASS-4 automated spraying system (equipped with TEEJET8002VS nozzles; developed by the National Engineering Research Center for Information Technology in Agriculture). Aboveground biomass was harvested 21 days post-treatment, oven-dried at 70 °C until constant weight, and then weighed to calculate dry weight inhibition rates. The experiment was conducted twice.

#### 4.2.2. Data Analysis

The median effective dose (GR_50_) for dry weight inhibition of different *Abutilon theophrasti* populations treated with glufosinate ammonium was calculated using a biphasic logistic nonlinear regression model in SigmaPlot 12.5 software, according to the following equation [46]:

(2) $$Y=C+\frac{D-C}{1+{\left(X∕GR_{50}\right)}^{b}}$$

where Y represents the dry weight inhibition rate of aboveground biomass post-treatment, D and C denote the upper and lower limits of the dose–response curve, respectively, X indicates the applied herbicide dose, GR_50_ corresponds to the median effective dose for dry weight inhibition of *Abutilon theophrasti,* and b represents the slope parameter.

#### 4.2.3. Sample Collection

Leaf tissue samples were collected from experimental plants at 0, 1, 2, 3, 5, and 7 days after treatment (DAT). Collected specimens were immediately flash-frozen in liquid nitrogen and subsequently maintained at −80 °C for biochemical analyses. Pre-treatment leaf samples were harvested as blank controls prior to herbicide application. Based on the manifestation of symptoms after administration, the samples collected on Day 3 post-treatment were selected for subsequent metabolomic analysis.

### 4.3. Sample Preparation and Extraction

#### 4.3.1. Dry Sample Extraction

Lyophilization was performed using a Scientz-100F freeze dryer (Ningbo Xinzhi Biotechnology Co., Ltd., Ningbo, China) to process biological specimens. The freeze-dried samples were homogenized into fine powder (30 Hz, 1.5 min) using a Retsch MM 400 ball mill (RETSCH Company, Hanau, Germany). Aliquots of precisely 50 mg of the powdered material were measured with an MS105DΜ analytical balance and extracted with a 1200 μL of ice-cold (−20 °C) methanol/water (70:30, *v*/*v*) internal standard solution, maintaining an extractant-to-sample ratio of 1200:50 (*v*/*w*). The mixture underwent intermittent vortex extraction (30 sec agitation at 30 min intervals for 6 cycles). Following centrifugation at 12,000× *g* for 3 min, the supernatant was filtered through a 0.22 μm nylon syringe filter and transferred to certified LC-MS vials for Ultra-Performance Liquid Chromatograph–Tandem Mass Spectrometry (UPLC-MS/MS) analysis.

#### 4.3.2. UPLC Conditions

The sample extracts were analyzed using an UPLC-ESI-MS/MS system (UPLC, ExionLC™ AD (SCIEX Company, Framingham, MA, USA), https://sciex.com.cn/, accessed on 14 November 2024) and Tandem Mass Spectrometry System (https://sciex.com.cn/, accessed on 14 November 2024). The analytical conditions were as follows, UPLC: column, Agilent SB-C18 (Agilent Company, Santa Clara, CA, USA) (1.8 µm, 2.1 mm × 100 mm). The mobile phase consisted of solvent A, pure water with 0.1% formic acid, and solvent B, and acetonitrile with 0.1% formic acid. The samples were measured using a gradient program, with the initial conditions of 95% A and 5% B. Within 9 min, a gradient to 5% A, 95% B was set, and a composition of 5% A, 95% B was maintained for 1 min. And the composition of 95% A, 5.0% B was adjusted within 1.1 min and maintained for 2.9 min. The flow rate was set at 0.35 mL/min and the column oven was set at 40 °C. The injection volume was 2 μL.

#### 4.3.3. ESI-Q TRAP-MS/MS

The operating parameters of the ESI source were established as follows: the source temperature was maintained at 500 °C; the ion spray voltage (IS) was set to 5500 V in positive ion mode and −4500 V in negative ion mode; ion source gas I (GSI), gas II (GSII), and curtain gas (CUR) were operated at 50, 60, and 25 psi, respectively; and the collision-activated dissociation (CAD) parameter was maintained at a high setting. QQQ scans were conducted as MRM experiments, with the collision gas (nitrogen) being set to medium. The declustering potential (DP) and collision energy (CE) for individual MRM transitions were determined with subsequent DP and CE optimization. For each period, a specific set of MRM transitions was monitored, based on the metabolites eluted during that period.

### 4.4. Data Analysis

#### 4.4.1. Principal Component Analysis

The implementation of unsupervised Principal Component Analysis (PCA) was carried out utilizing the prcomp function within the R programming environment (www.r-project.org, accessed on 14 November 2024). Prior to the application of unsupervised PCA, the dataset underwent scaling to unit variance.

#### 4.4.2. Hierarchical Cluster Analysis and Pearson’s Correlation Coefficients

The results of hierarchical cluster analysis (HCA) conducted on samples and metabolites were presented as heatmaps incorporating dendrograms. Concurrently, Pearson’s correlation coefficients (PCC) between samples were computed using the cor function in R and were displayed exclusively in heatmap format. Both HCA and PCC analyses were performed utilizing the R package ComplexHeatmap (2.9.4). For the HCA visualization, normalized signal intensities of metabolites (achieved through unit variance scaling) were represented using a color spectrum.

#### 4.4.3. Differential Metabolites Selected

For comparative analysis of two groups, a set of differential metabolites was identified based on VIP thresholds (VIP > 1) and absolute logarithmic fold change criteria (|Log2FC| ≥ 1.0). In multi-group comparative analyses, differential metabolites were selected according to the VIP values (VIP > 1) and statistically significant *p*-values (*p*-value < 0.05 derived from ANOVA). The VIP values were derived from the OPLS-DA model, which was constructed using the R package MetaboAnalystR and contained both score visualizations and permutation diagnostic plots. Prior to OPLS-DA modeling, the dataset was processed through logarithmic transformation (base 2), followed by mean-centering normalization. To mitigate overfitting risks, a permutation assessment comprising 200 iterative permutations was conducted.

#### 4.4.4. KEGG Annotation and Enrichment Analysis

The identified metabolites were annotated employing the KEGG Compound database (http://www.kegg.jp/kegg/compound/, accessed on 8 July 2024). Subsequently, these annotated metabolites were mapped onto the KEGG Pathway database (http://www.kegg.jp/kegg/pathway.html, accessed on 8 July 2024).

## 5. Conclusions

The widely targeted metabolomics analysis revealed significant perturbations in multiple metabolic pathways following glufosinate ammonium treatment in *Abutilon theophrasti*, particularly involving D-amino acid metabolism, Biosynthesis of amino acids, and Arginine–proline metabolism. These findings provide novel insights into the potential metabolic mechanisms underlying weed response to herbicide stress.

We identified three significant pathways in the metabolism of susceptible and tolerant *Abutilon theophrasti* to glufosinate ammonium by widely targeted metabolomics, which may influence the level of tolerance to glufosinate ammonium. The D-amino acid metabolism may be the potential interference with herbicide–target interactions. The Biosynthesis of amino acids may play a role of metabolic rerouting to resist glufosinate ammonium inhibition. And Arginine–proline metabolism is mainly responsible for ROS mitigation and osmotic adjustment for cellular homeostasis. Therefore, these three metabolic pathways may form an integrated detoxification network, where D-amino acid metabolism provides immediate stress signaling, Biosynthesis of amino acids restores primary metabolism, and Arginine–proline metabolism alleviates downstream oxidative damage.

## Figures and Tables

**Table 1:** The doses of glufosinate ammonium used for determining the resistance ratio of *Abutilon theophrasti*.

Populations	Glufosinate Ammonium (g a.i. ha^−1^)
S	0, 23.4375, 46.875, 93.75, 187.5, 375, 750 *
SR	0, 93.75, 187.5, 375, 750 *, 1500, 3000

750 * is the active ingredient amount of the recommended field dose.

## Data Availability

The original contributions presented in this study are included in the article/Supplementary Materials. Further inquiries can be directed to the corresponding author.

## Supplementary Materials

The following supporting information can be downloaded at: https://www.mdpi.com/article/10.3390/plants14131994/s1.
