# Supplementary figures and images for: Widely Targeted Metabolomics Reveals Metabolic Divergence in Abutilon theophrasti Populations Under Glufosinate Ammonium Treatment
Source: Plants (Basel). 2025 Jun 30;14(13):1994. doi: 10.3390/plants14131994 (PMC12251701; doi:10.3390/plants14131994)

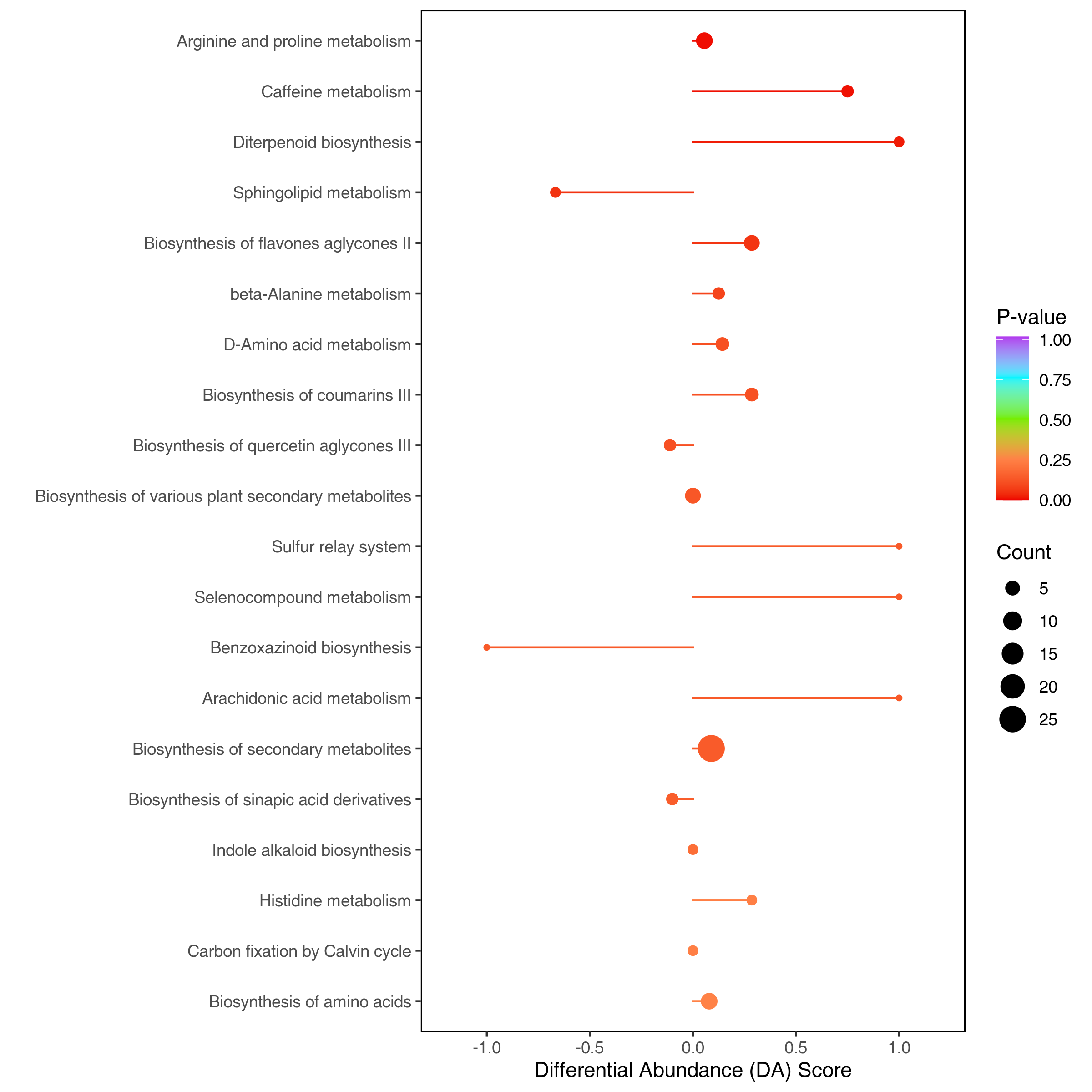

Supplement: Supplementary file 1 [file plants-14-01994-s001.zip › Figure S10——R-Treat_vs_S-Treat_KEGG_DA_score_P-value.png]

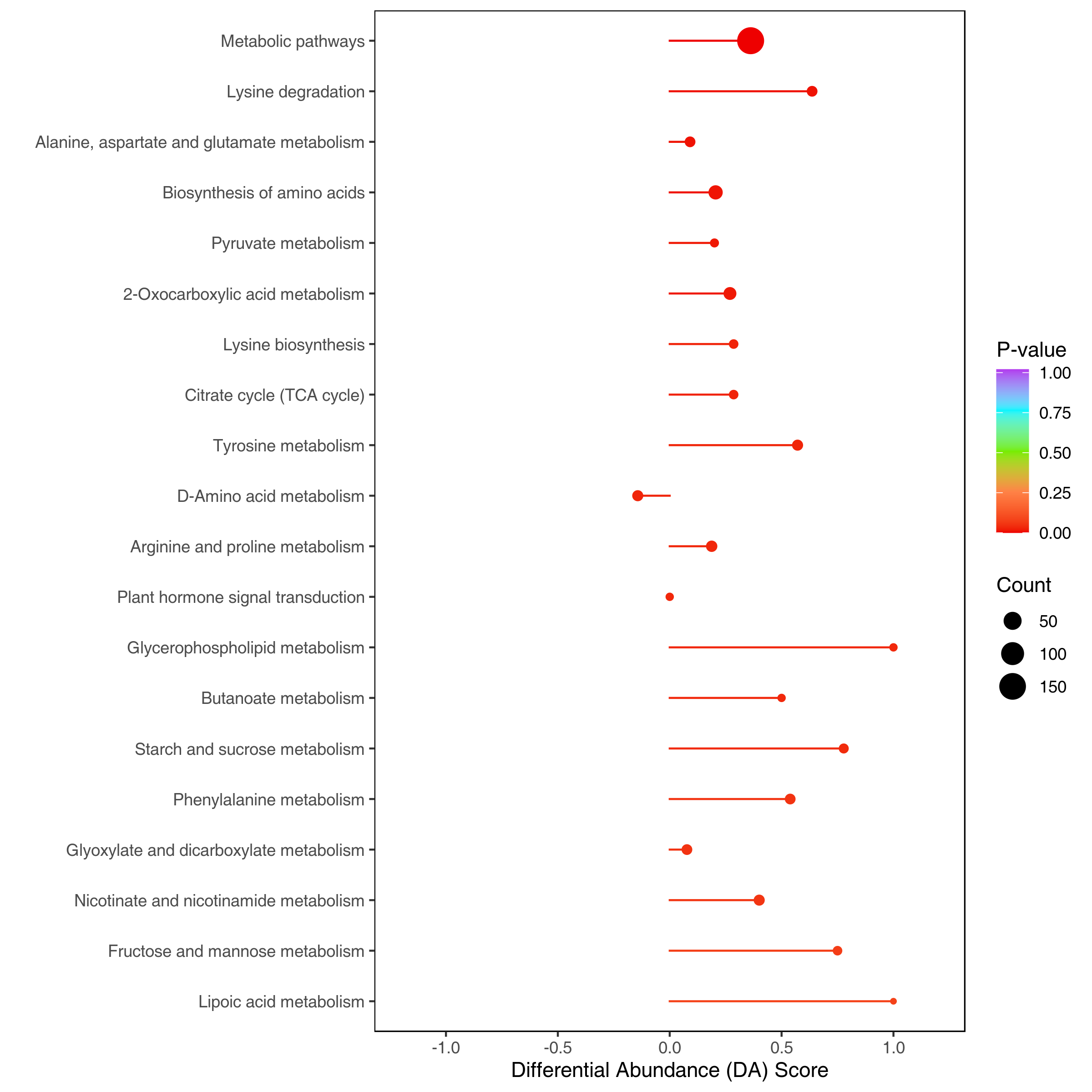

Supplement: Supplementary file 1 [file plants-14-01994-s001.zip › Figure S11——R-Treat_vs_R-CK_KEGG_DA_score_P-value.png]

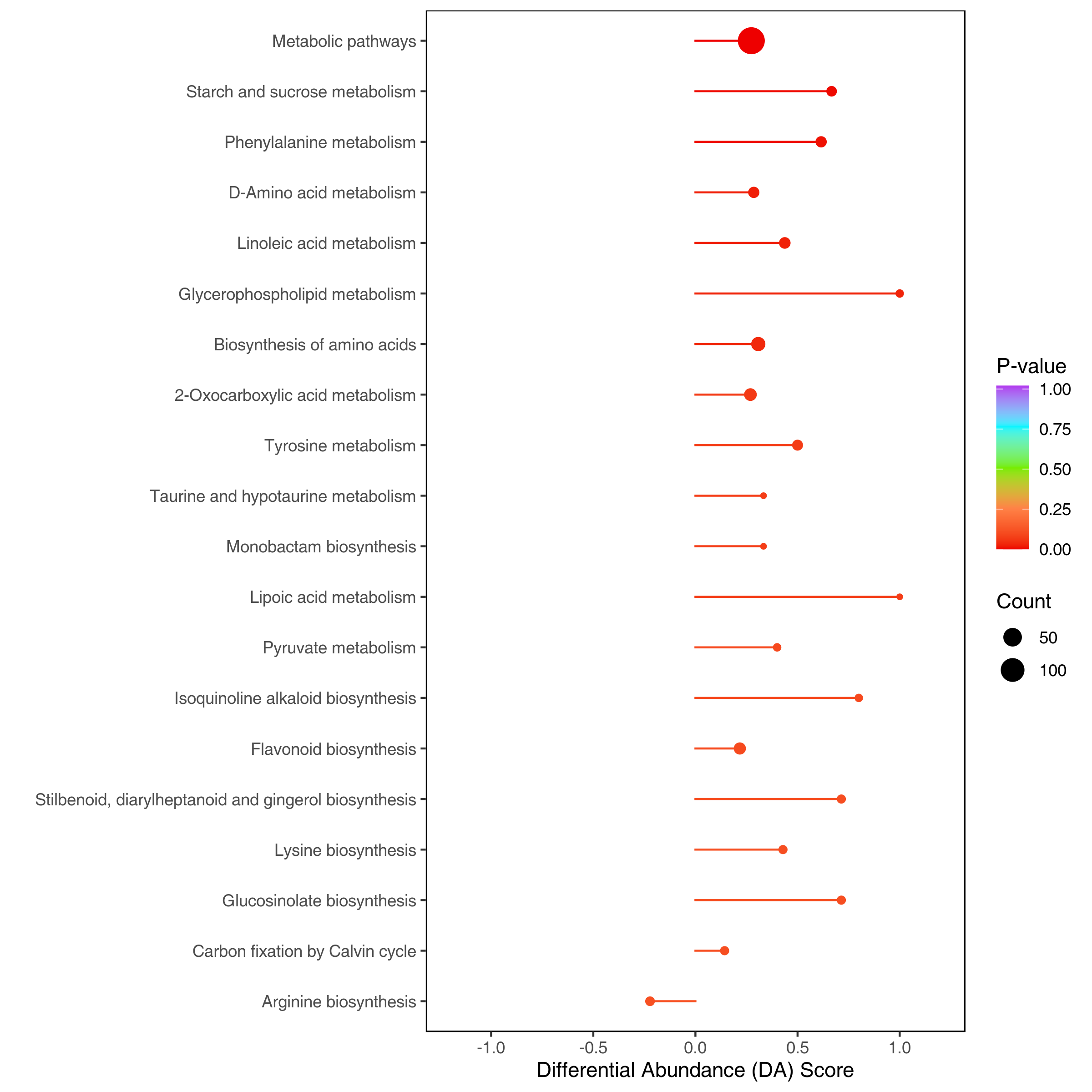

Supplement: Supplementary file 1 [file plants-14-01994-s001.zip › Figure S12——S-Treat_vs_S-CK_KEGG_DA_score_P-value.png]

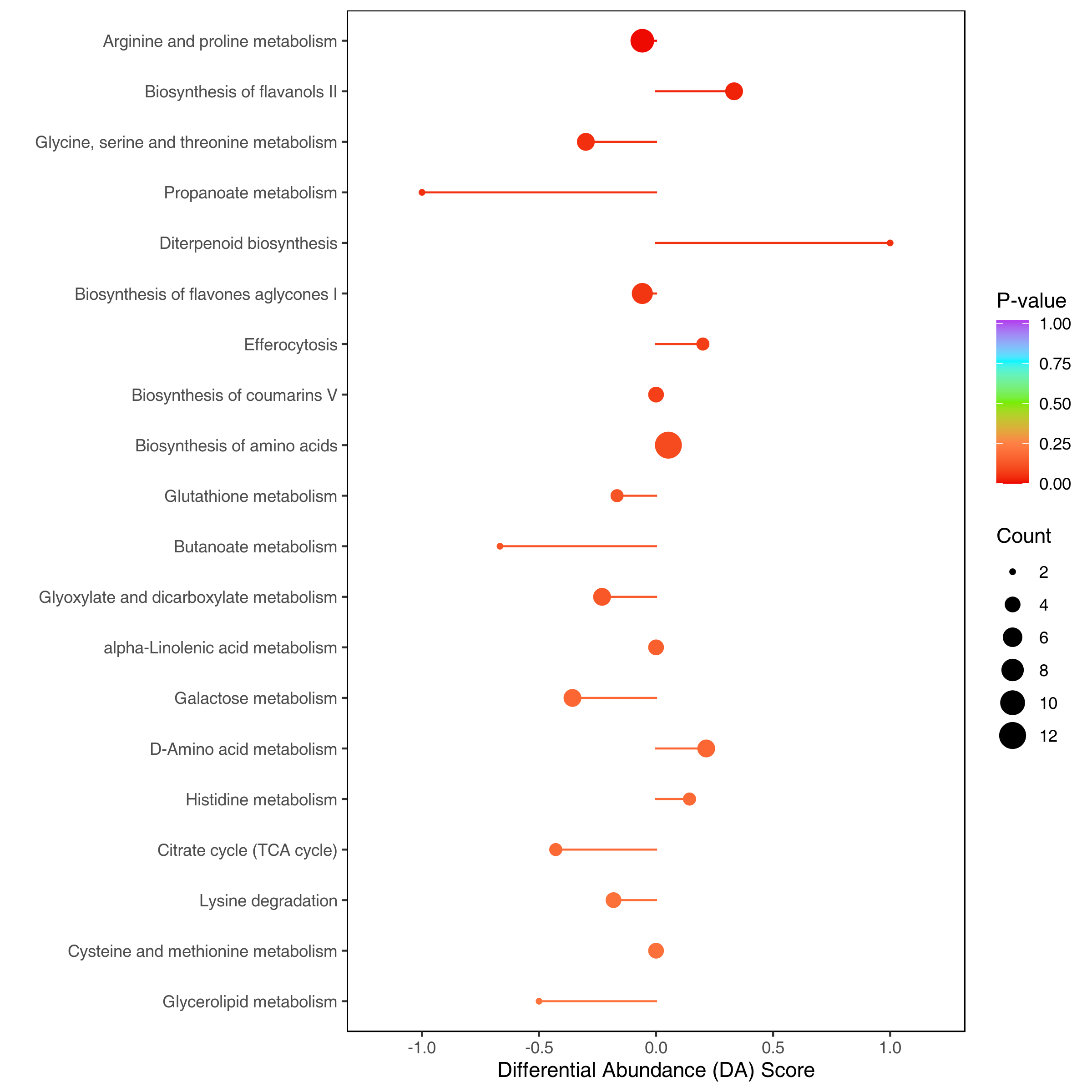

Supplement: Supplementary file 1 [file plants-14-01994-s001.zip › Figure S13——R-CK_vs_S-CK_KEGG_DA_score_P-value.png]

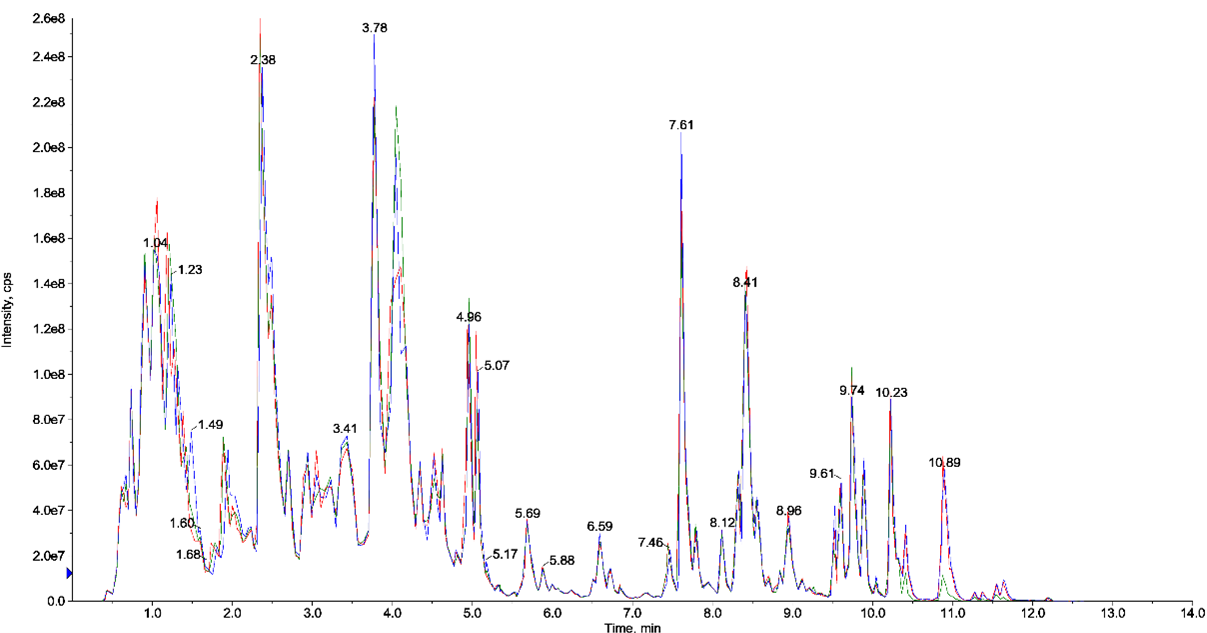

Supplement: Supplementary file 1 [file plants-14-01994-s001.zip › Figure S1(A)——The total ion current profiles from three quality control (MIX) samples.png]

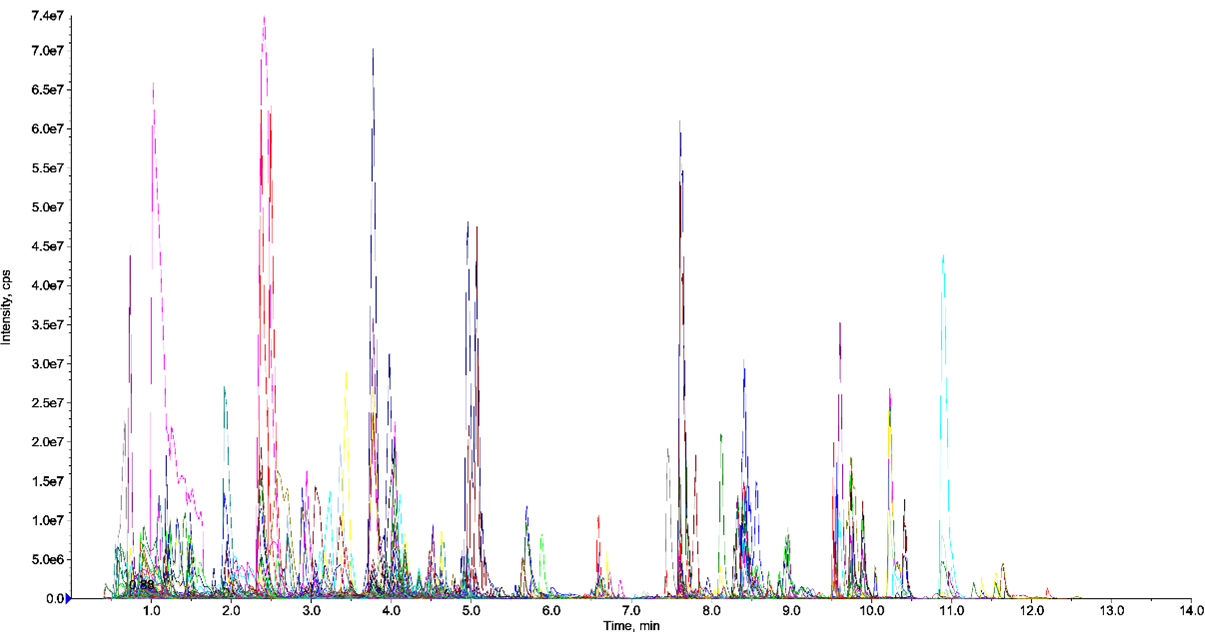

Supplement: Supplementary file 1 [file plants-14-01994-s001.zip › Figure S1(B)——The multiple reaction monitoring (MRM)-based metabolite detection multi-peak chromatogram.png]

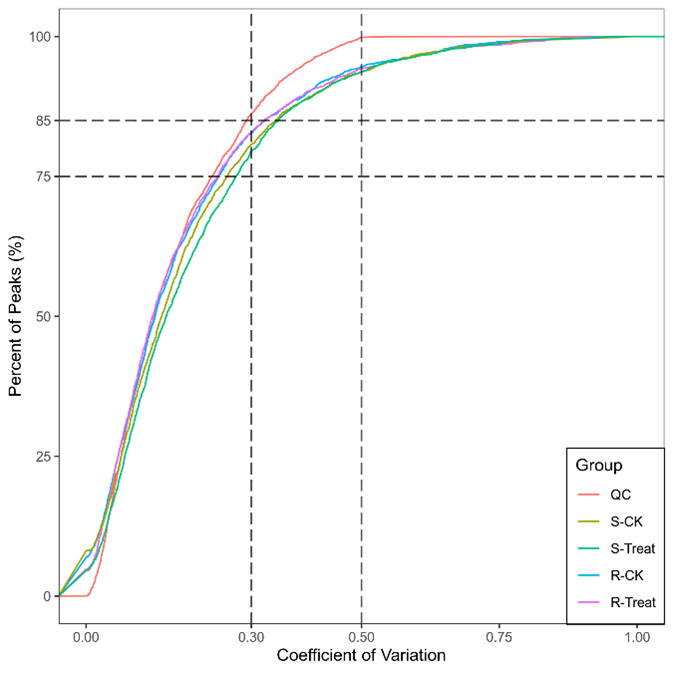

Supplement: Supplementary file 1 [file plants-14-01994-s001.zip › Figure S2——CV distribution of each group.png]

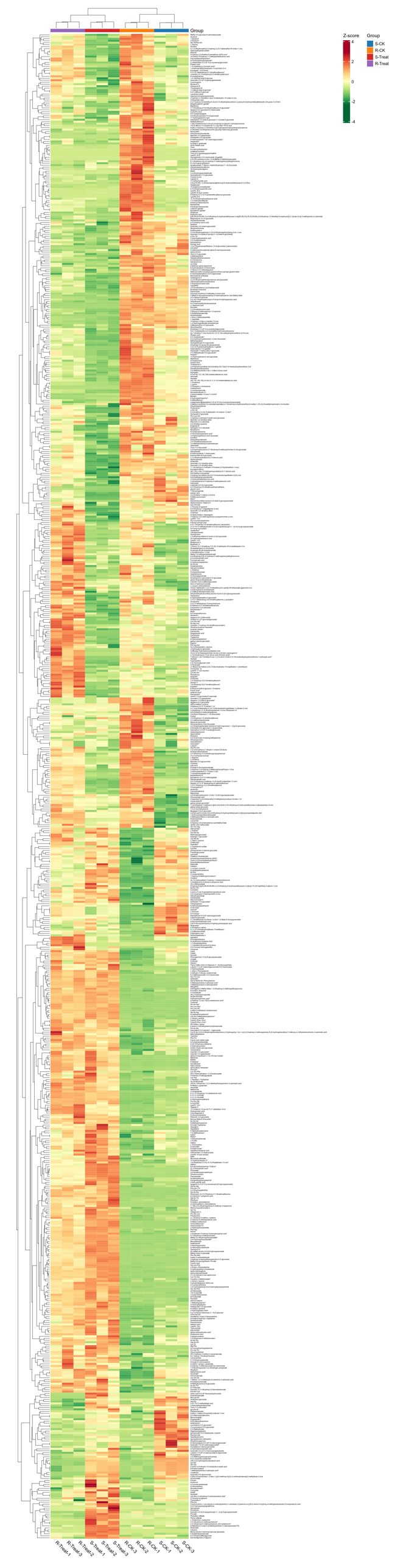

Supplement: Supplementary file 1 [file plants-14-01994-s001.zip › Figure S3——S-CK_vs_R-CK_vs_S-Treat_vs_R-Treat_heatmap_col-row_cluster_Compounds.png]

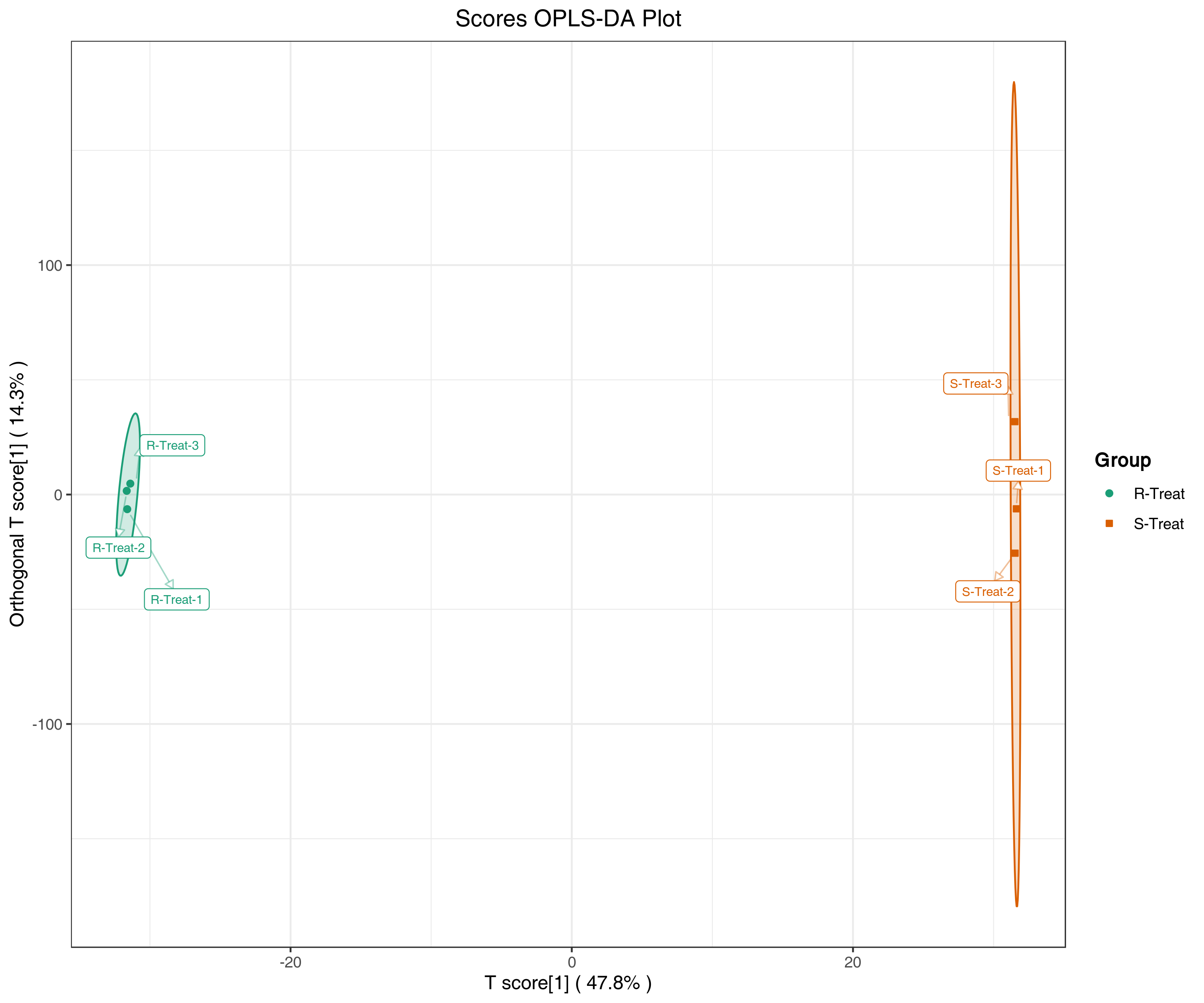

Supplement: Supplementary file 1 [file plants-14-01994-s001.zip › Figure S4 (A)——R-Treat_vs_S-Treat_OPLS-DA_scorePlot.png]

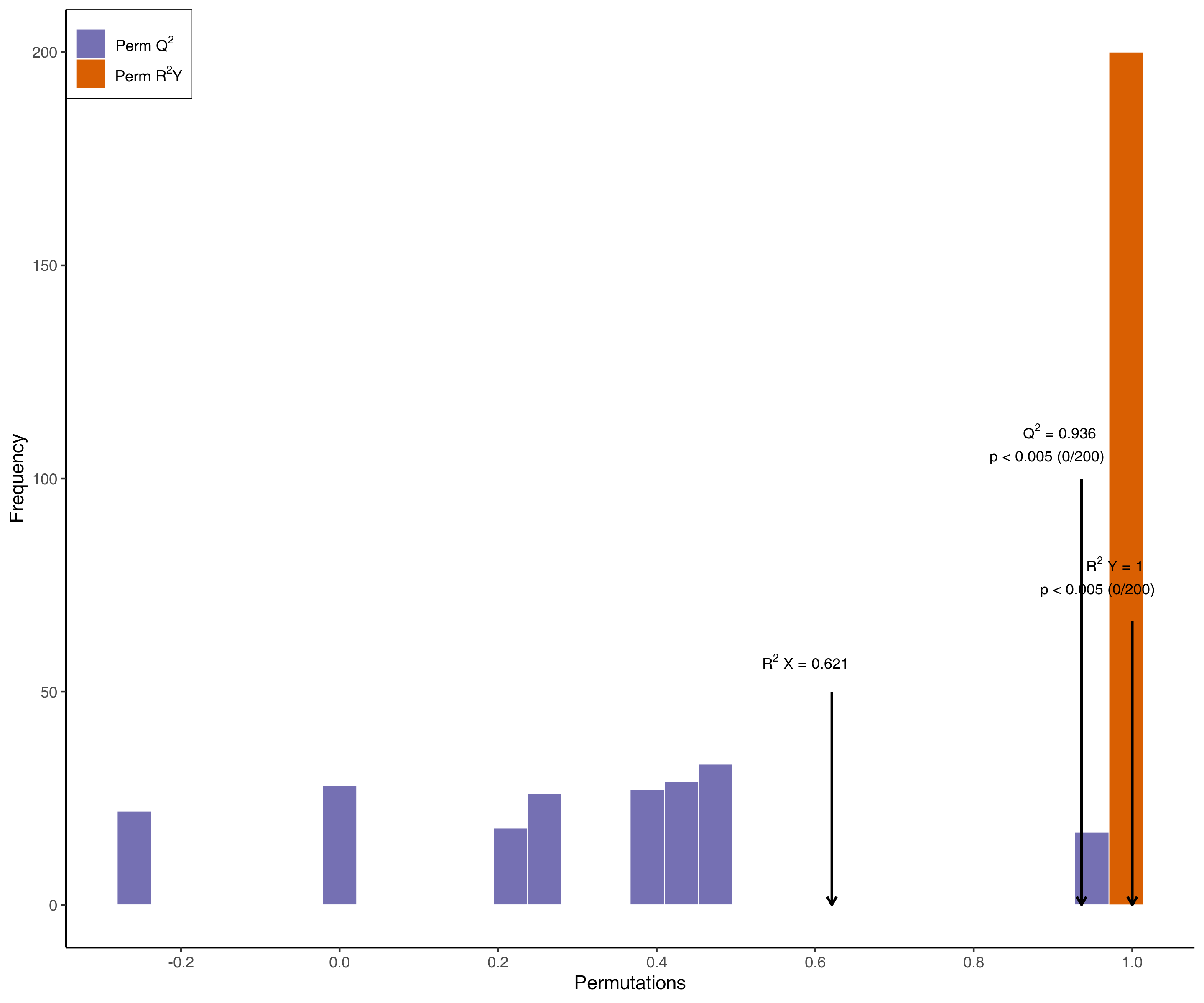

Supplement: Supplementary file 1 [file plants-14-01994-s001.zip › Figure S4 (B)——R-Treat_vs_S-Treat_OPLS-DA_permutation.png]

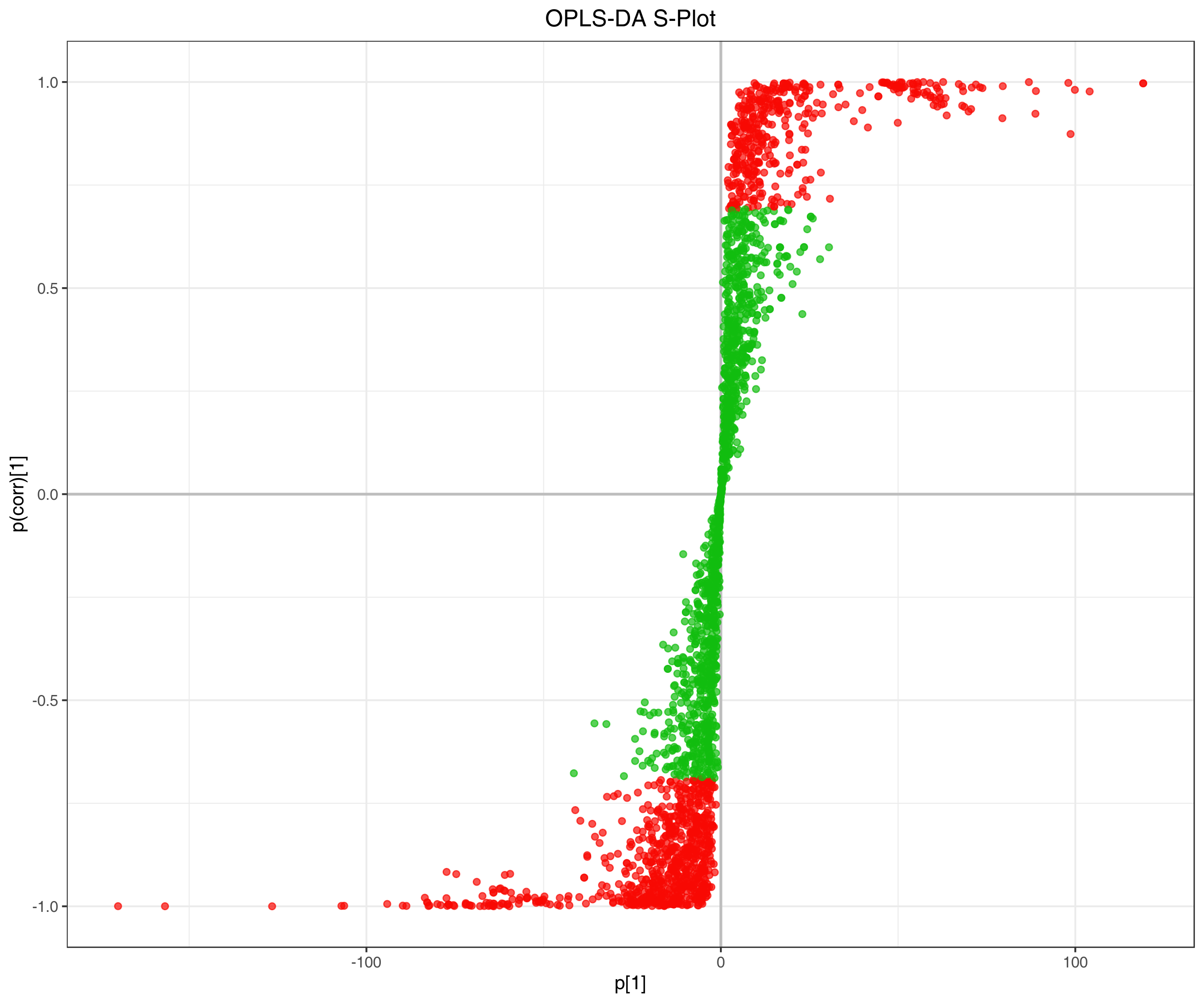

Supplement: Supplementary file 1 [file plants-14-01994-s001.zip › Figure S5——R-Treat_vs_S-Treat_OPLS-DA_SPlot.png]

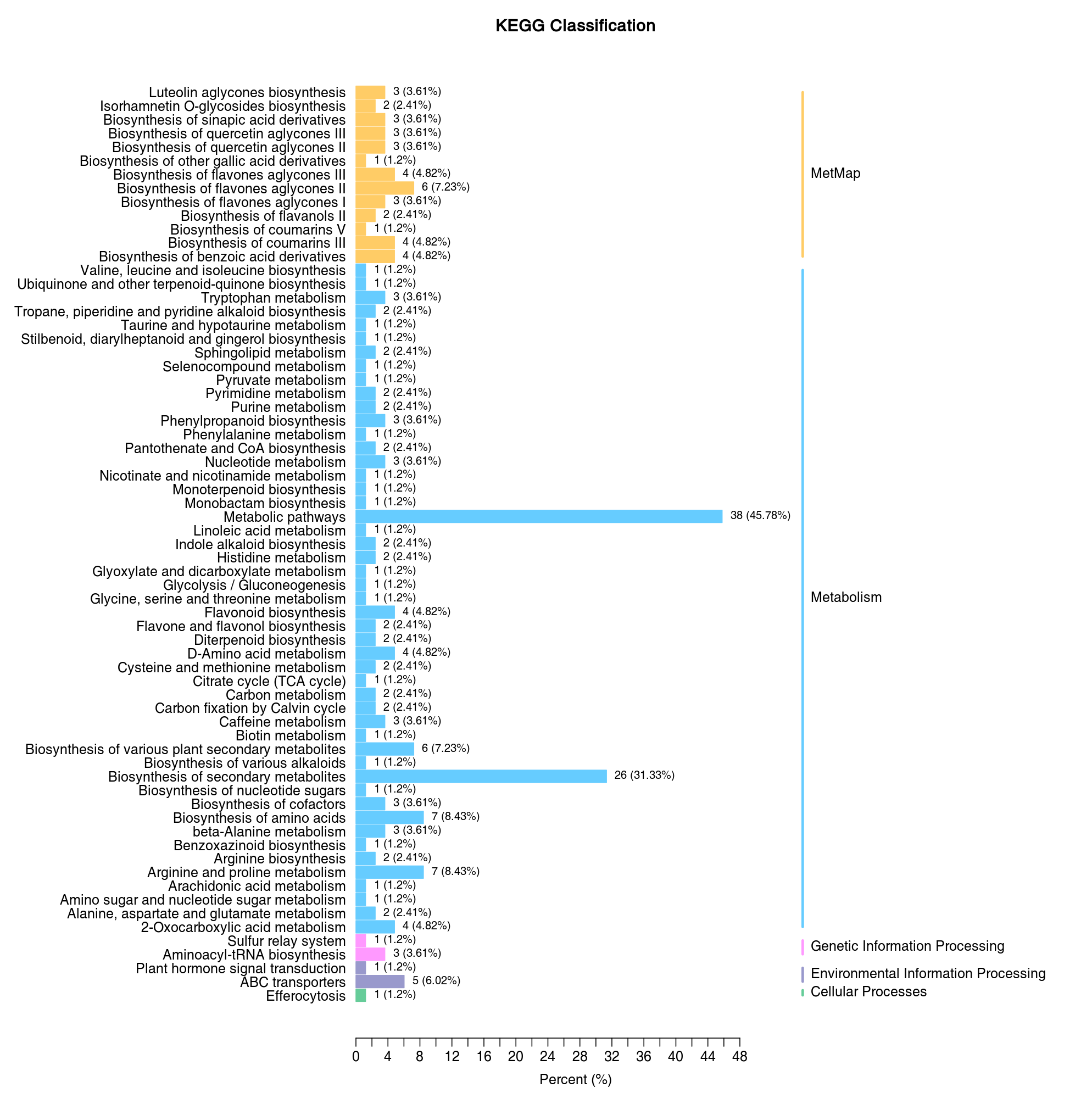

Supplement: Supplementary file 1 [file plants-14-01994-s001.zip › Figure S6——R-Treat_vs_S-Treat_KEGG_barplot.png]

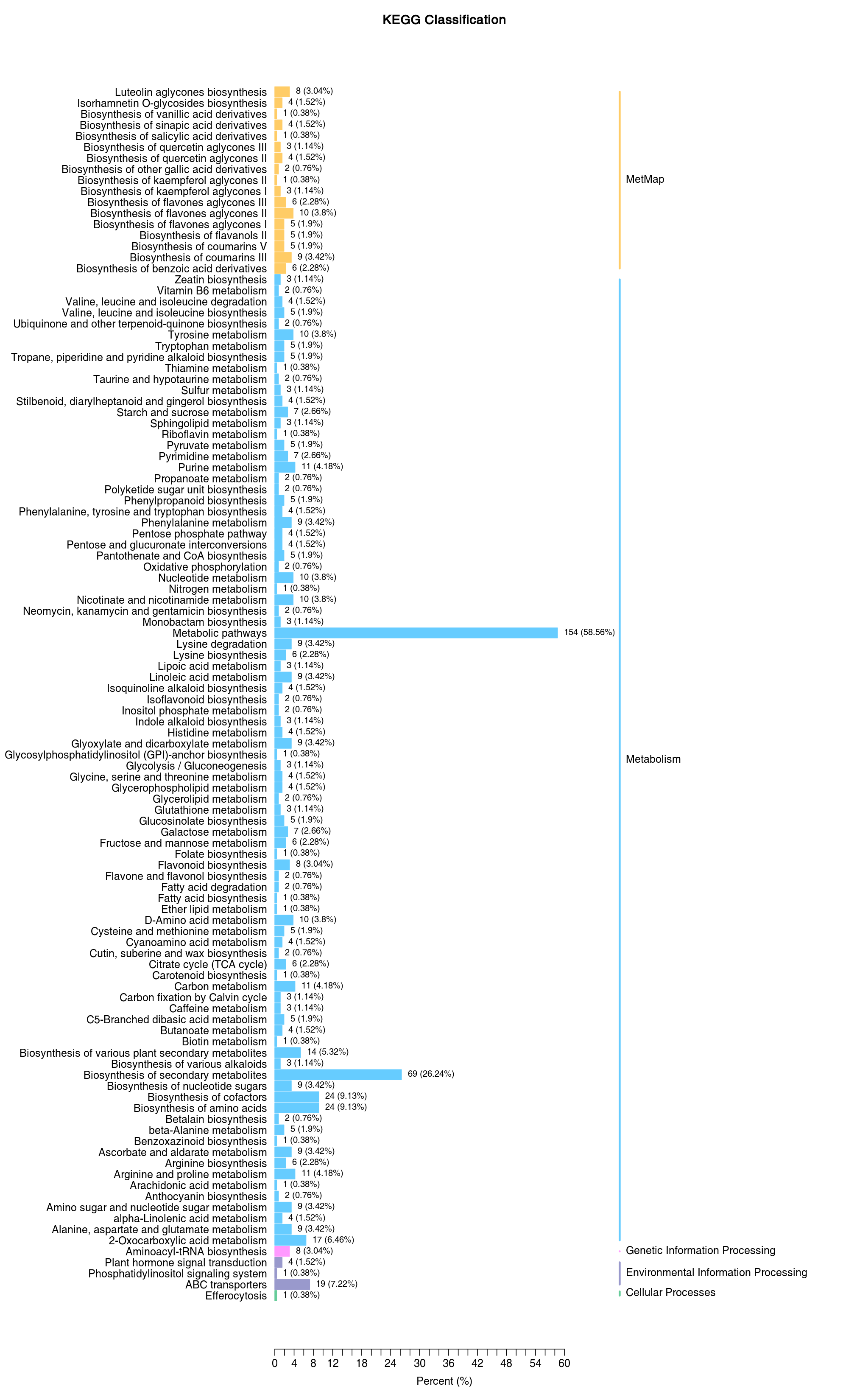

Supplement: Supplementary file 1 [file plants-14-01994-s001.zip › Figure S7——R-Treat_vs_R-CK_KEGG_barplot.png]

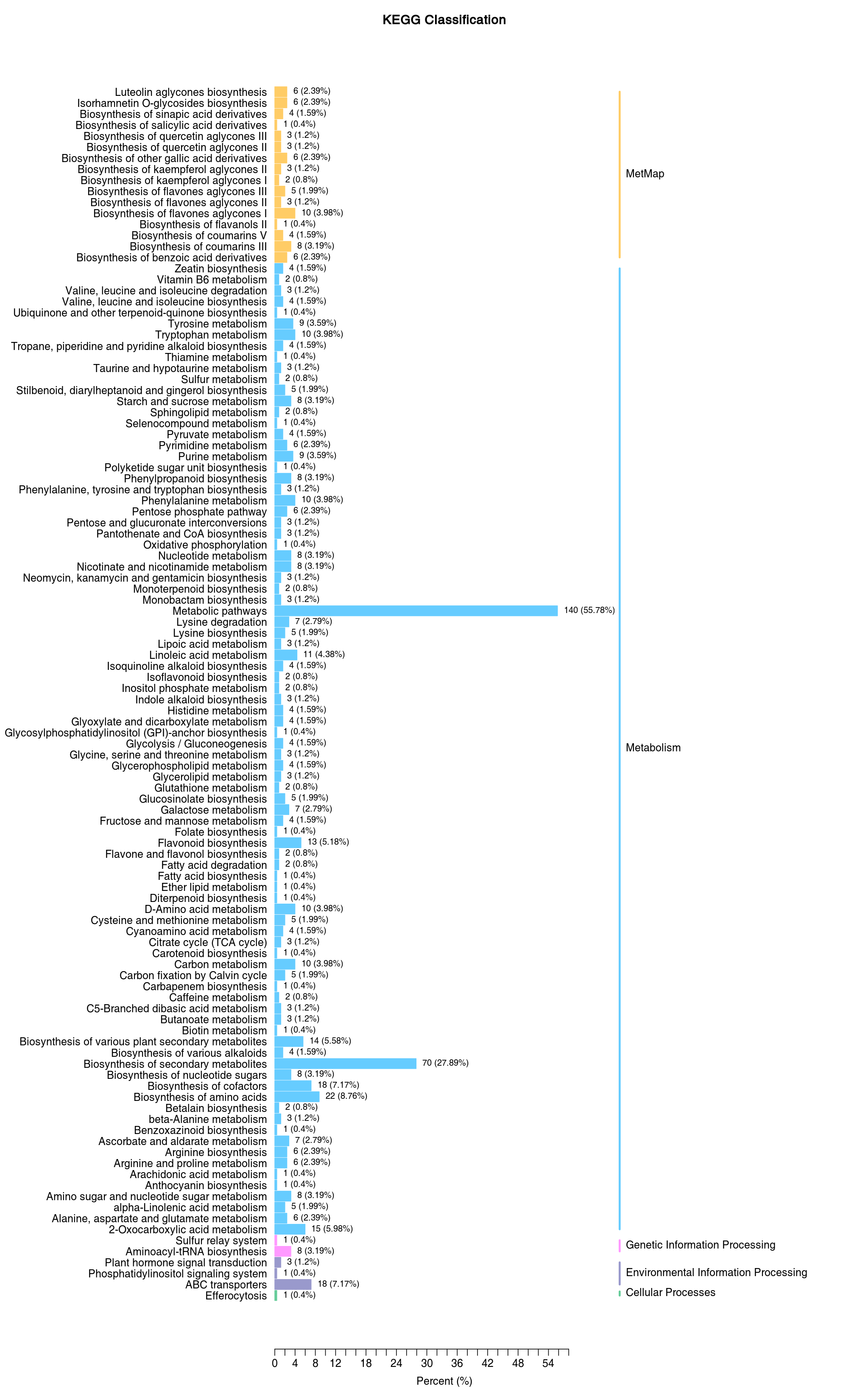

Supplement: Supplementary file 1 [file plants-14-01994-s001.zip › Figure S8——S-Treat_vs_S-CK_KEGG_barplot.png]

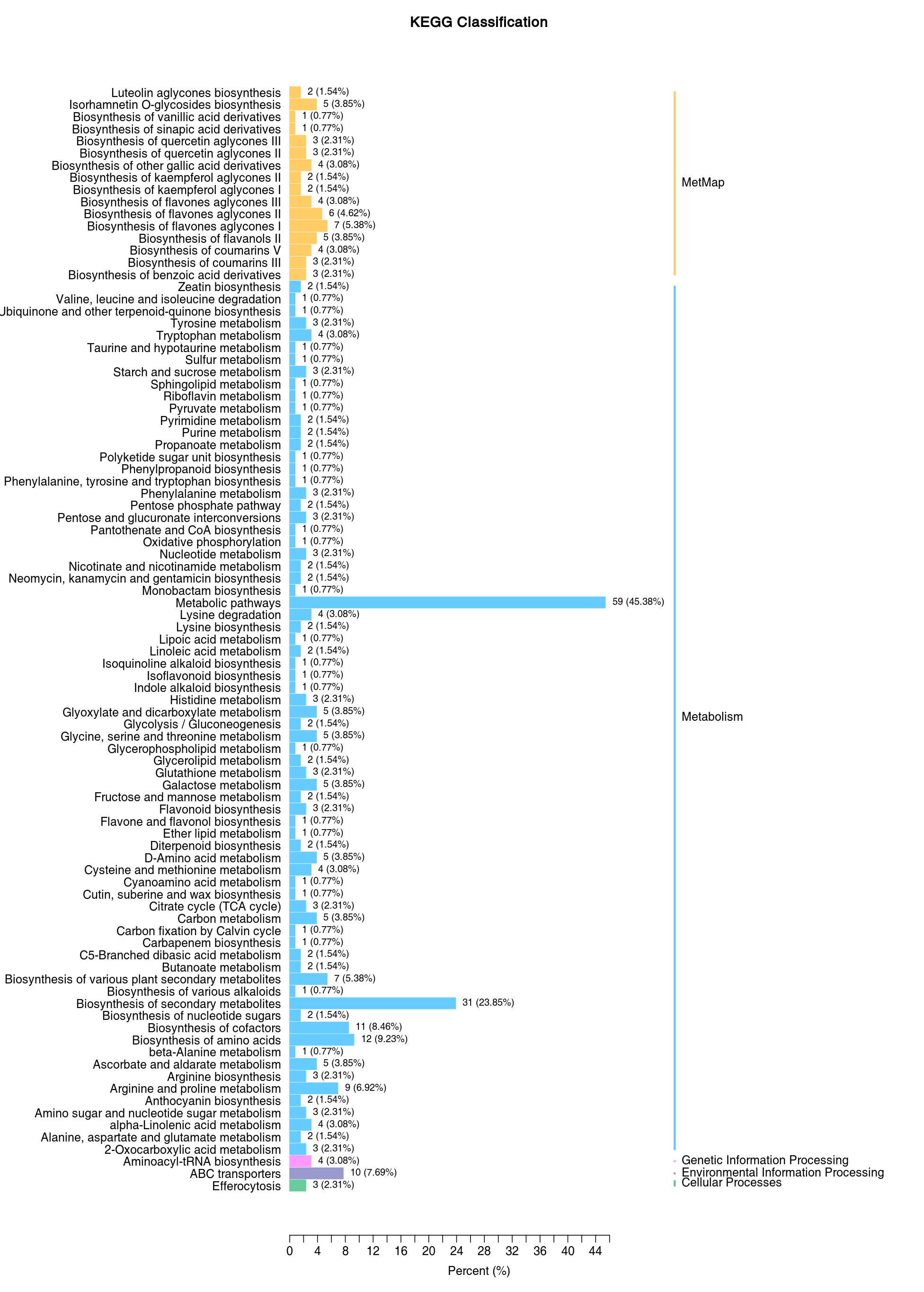

Supplement: Supplementary file 1 [file plants-14-01994-s001.zip › Figure S9——R-CK_vs_S-CK_KEGG_barplot.png]
